# CSF Secretion Is Not Altered by NKCC1 Nor TRPV4 Antagonism in Healthy Rats

**DOI:** 10.3390/brainsci11091117

**Published:** 2021-08-24

**Authors:** Steven W. Bothwell, Daniel Omileke, Adjanie Patabendige, Neil J. Spratt

**Affiliations:** 1 The School of Biomedical Sciences and Pharmacy, The University of Newcastle, Callaghan, NSW 2308, Australia; steven.bothwell@uon.edu.au (S.W.B.); daniel.omileke@uon.edu.au (D.O.); 2 Hunter Medical Research Institute, New Lambton Heights, NSW 2305, Australia; 3 Institute of Infection, Veterinary & Ecological Sciences, University of Liverpool, Wirral CH64 7TE, UK; 4 Hunter New England Local Health District, New Lambton Heights, NSW 2305, Australia

**Keywords:** cerebrospinal fluid, choroid plexus, NKCC1, TRPV4, intracranial pressure, loop diuretics

## Abstract

Background: Cerebrospinal fluid (CSF) secretion can be targeted to reduce elevated intracranial pressure (ICP). Sodium-potassium-chloride cotransporter 1 (NKCC1) antagonism is used clinically. However, supporting evidence is limited. The transient receptor potential vanilloid-4 (TRPV4) channel may also regulate CSF secretion and ICP elevation. We investigated whether antagonism of these proteins reduces CSF secretion. Methods: We quantified CSF secretion rates in male Wistar rats. The cerebral aqueduct was blocked with viscous mineral oil, and a lateral ventricle was cannulated. Secretion rate was measured at baseline and after antagonist administration. Acetazolamide was administered as a positive control to confirm changes in CSF secretion rates. Results: Neither NKCC1, nor TRPV4 antagonism altered CSF secretion rate from baseline, *n* = 3, t(2) = 1.14, *p* = 0.37, and *n* = 4, t(3) = 0.58, *p* = 0.6, respectively. Acetazolamide reduced CSF secretion by ~50% across all groups, *n* = 7, t(6) = 4.294, *p* = 0.005. Conclusions: Acute antagonism of NKCC1 and TRPV4 proteins at the choroid plexus does not reduce CSF secretion in healthy rats. Further investigation of protein changes and antagonism should be explored in neurological disease where increased CSF secretion and ICP are observed before discounting the therapeutic potential of protein antagonism at these sites.

## 1. Introduction

Intracranial pressure (ICP) elevation is reported in several neurological conditions including hydrocephalus [1,2], idiopathic intracranial hypertension [3], traumatic brain injury [4,5,6], subarachnoid haemorrhage [7,8,9], and stroke [10,11,12,13,14,15]. Elevated ICP can cause secondary brain injury and death, so should be monitored and managed as a priority for patients with neurological disease or injury [16,17]. Current treatments to manage ICP include: elevation of the head, hyperventilation, the use of osmotic agents, drainage of cerebrospinal fluid (CSF) through lumbar puncture or via an external ventricular drain, and/or decompressive craniectomy–usually considered a last resort [16,18]. Therapies targeting volume reduction of brain tissue, cerebral blood volume, and CSF volume are often used to reduce ICP rise, e.g., managing oedema with osmotherapy, managing venous drainage of cerebral blood with neck positioning, and reducing CSF secretion with inhibitors of ion channels and enzymes at the choroid plexus, the primary site of CSF secretion, such as acetazolamide, bumetanide, and furosemide [18].

Pharmaceuticals for managing ICP are limited. Acetazolamide reduces CSF secretion by ~50% by inhibiting carbonic anhydrase, an intracellular enzyme in the choroid plexus epithelia contributing to HCO_3_^−^ accumulation and has been shown to rapidly decrease ICP in healthy rats [19,20]. Acetazolamide treatment has also been shown to prevent ICP spikes after intracerebral haemorrhage, which can prevent reduced cerebral blood flow and herniation [21]. It is often prescribed for the treatment of chronically elevated ICP in idiopathic intracranial hypertension and can be used for managing acutely elevated ICP in intensive care [22]. However, the effectiveness of acetazolamide treatment is unclear, with only modest reductions in ICP observed in patients [23]. Further, side effects like fatigue, nausea, vomiting and abdominal pain can be distressing for patients. Bumetanide and similar loop diuretics, such as furosemide, target the sodium-potassium-chloride cotransporter 1 (NKCC1) and were previously proposed as potential therapeutics for managing ICP [24,25,26]. The choroid plexus located in the ventricular system of the brain secretes 80–90% of CSF, while fluid transport at the blood brain barrier makes up the remaining 10–20% [27,28,29,30]. Na^+^ and Cl^−^ are the predominant ions present in CSF [31]. NKCC1 is highly expressed on the apical membrane of choroid plexus epithelial cells and active transport of Na^+^, K^+^, and Cl^−^ is an important driver of CSF secretion. The resulting osmotic gradient allows passive water transport into the ventricles via the aquaporin 1 (AQP1) water transporter [32]. AQPs are involved in ion and water transport and are expressed in most tissues, including tissues in the CNS [33]. AQP1, 4, and 9 are the predominant subtype expressed in the brain and AQP1 is highly expressed at the choroid plexus [34]. Clinical and in vivo animal studies have demonstrated that bumetanide can reduce ICP in combination with osmotherapy or acetazolamide [31,35,36,37,38,39]. However, there is limited evidence of the effects of NKCC1 inhibition alone on CSF secretion. One study in dogs found that intraventricular bumetanide infusion reduced CSF secretion [31]. Another study reported a ~48% decrease in CSF secretion in mice after intraventricular bumetanide administration [40]. Furosemide produced a modest reduction in CSF secretion in rabbits but there was no reduction in response to bumetanide [19]. These studies utilised tracer dilution techniques after intraventricular infusion to determine CSF secretion, which may not provide an accurate representation, as variations to CSF flow or perfusion into the brain parenchyma may influence observations. A direct CSF secretion measurement technique was previously described, which offers real-time quantification of CSF secretion rates in response to antagonists [41]. This technique would be advantageous for determining the effect of bumetanide on CSF secretion. The past two decades have seen a shortage of novel research to support the role of NKCC1 antagonists in reducing CSF secretion and ICP. The clinical use of bumetanide and similar loop diuretics for ICP rise after neurological injury is not supported by clinical guidelines, likely due to the absence of evidence for their effectiveness [42,43]. Therefore, the need for validation of bumetanide or the implementation of novel pharmacological therapies for managing ICP rise is immediate.

The transient receptor potential vanillioid-4 (TRPV4) channel is highly expressed at the apical membrane of the choroid plexus and is also highly expressed in renal tissue [44,45]. Activation of TRPV4 increases transepithelial permeability and increases water efflux across the blood-CSF-barrier (BCSFB) through interactions with anoctamin 1 [46]. Recent evidence suggests that it may be involved in the regulation of CSF secretion by controlling transepithelial water permeability at the choroid plexus in response to multiple stimuli [47]. TRPV4 is activated by several stimuli like hypotonic stress [48], temperature [49], mechanical stress [50], and ligand binding [51], so it presents as a potential hub protein for the regulation of CSF secretion. TRPV4 is also upregulated and activated in brain parenchyma after ischaemic stroke and traumatic brain injury [52,53,54]. Further, a recent preclinical study found that chronic TRPV4 antagonism effectively ameliorates symptoms of hydrocephalus (cranial expansion and ventriculomegaly) in a genetic model of the disease [55]. These findings suggest that TRPV4 antagonism could be a novel target for reducing ICP rise in neurological disease or injury. Currently, in vivo evidence for the role of TRPV4 in CSF secretion is absent.

We aimed to determine whether antagonism of NKCC1 or TRPV4 proteins reduces CSF secretion acutely in healthy rats. The intention of this study was to build an evidence base to support the role of NKCC1 and TRPV4 as targets for managing CSF secretion before investigating their therapeutic potential for managing ICP in neurological disease and injury. We anticipated that antagonism of NKCC1 would reduce CSF secretion due to the existing clinical applications of loop diuretics and some pre-clinical studies that support the use of bumetanide for managing ICP. We suspected that TRPV4 antagonism would also result in reduced CSF secretion due to its success in ameliorating symptoms of CSF accumulation in hydrocephalus. Antagonism of NKCC1 nor TRPV4 reduced CSF secretion rate in healthy rats, suggesting that neither protein is likely to act as a sole regulator of CSF secretion under normal conditions. However, we cannot discount these proteins as targets of CSF secretion and ICP rise in neurological disease or injury. Further work must explore responses to antagonism under pathological conditions.

## 2. Materials and Methods

### 2.1. Experimental Design

**Figure 1:**
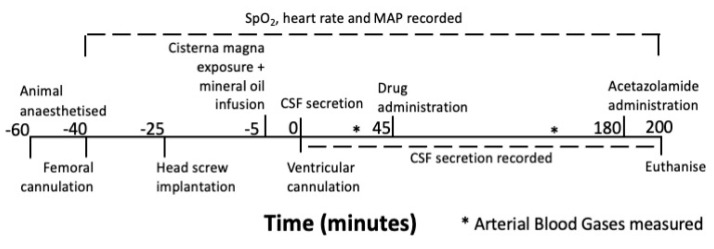
Experimental timeline. Oxygen saturation (SpO_2_), heart rate, and mean arterial pressure (MAP) were recorded from −40 to 200 min. CSF secretion was recorded from 0 to 200 min after mineral oil infusion via cisterna magna and ventricular cannulation. Bumetanide, RN1734, or DMSO was administered at 45 min and acetazolamide administered at 180 min. Recording was ceased at 200 min and the animal was euthanised. * Arterial Blood Gases were measured at 30 and 150 min.

The experimental timeline is outlined in Figure 1. Physiological variables were recorded throughout the procedure. Blood pressure and heart rate monitoring commenced following cannulation of the femoral artery. A screw was placed over the left lateral ventricle as a guide for ventricular cannulation. The cisterna magna was exposed and punctured to allow for cannulation and blockade of the cerebral aqueduct using mineral oil. The cannula was fixed in place using a sealant. Next, the left ventricle was cannulated, and CSF flow was observed along the silastic tubing. Baseline CSF secretion rate was recorded from 0 to 45 min before drug administration at 45 min. The antagonist-response recording period was from 45 to 180 min. Acetazolamide was administered at 180 min to confirm that true CSF secretion was observed (expecting CSF secretion to drop by ~50%). A blood sample was taken from the femoral cannula at 30 and 150 min for arterial blood gas measurement. Recording was ceased at 200 min and the animal was euthanised. We confirmed the cerebral aqueduct was adequately blocked with mineral oil by infusing Evans blue dye into the lateral ventricle after the recording period and found no dye beyond the third ventricle.

The TRPV4 antagonist, RN1734, in dimethylsulfoxide (DMSO), was administered intraperitoneally as previously described [55]. Although this method is not the most efficacious route of delivery, it is far less invasive than intraventricular administration and is more clinically relevant. We used 10 mg/kg of RN1734 as we expected this dose to produce an adequate response, considering 4 mg/kg is enough to reduce symptoms of hydrocephalus in rats and 5 mg/kg reduces TRPV4 expression in the CNS [55,56]. Further, we checked for changes to CSF secretion rate after administration of DMSO and confirmed that DMSO had no effect. We chose to administer 10 mg/kg of bumetanide. Doses as low as 0.2 mg/kg and as high as 50 mg/kg, administered intraventricularly and intraperitoneally, respectively, have previously elicited effects [19,57]. Bumetanide can be detected in brain tissue after systemic administration of a 10 mg/kg dose, which is enough to induce NKCC1 inhibition [58,59]. To our knowledge, there is no available data of dose-response for the effect of bumetanide on CSF secretion; therefore, we aimed to start with a supramaximal acute dose before determining the lowest effective dose.

### 2.2. Animals

Procedures were carried out on male outbred Wistar rats aged between 12–16 weeks and weighing between 265–330 g (*n* = 17). All experimental animal procedures used in this project were approved by the Animal Care and Ethics Committee of the University of Newcastle (A-2013-343 & A-2020-003).

Animals were excluded from experimentation if CSF flow along the ventricular cannula did not occur or was inconsistent during baseline recordings. Animals were assigned to treatment groups prior to intervention. Blinding was not carried out due to the exploratory nature of these experiments.

### 2.3. Anaesthesia and Monitoring

Rats were anaesthetised with isoflurane (5% induction, 1.75–2.5% maintenance) in 50:50% N_2_:O_2_. Incision sites were subcutaneously injected with 2 mg/kg 0.05% Bupivacaine (Pfizer, Sydney, Australia). Core body temperature was regulated via a thermocoupled rectal probe (RET-2, Physitemp Instruments Inc, Clifton, NJ, USA) and heat mat.

A catheter was inserted into the saphenous branch of the femoral artery and was used to obtain mean arterial blood pressure and heart rate. Blood samples (0.1 mL) were taken from the catheter for arterial blood gas and pH measurements using a fast blood analyser (i-STAT 1; Abbott, Australia). A similar catheter was placed into the femoral vein for infusion of acetazolamide. Oxygen saturation and respiration were monitored throughout procedures.

### 2.4. Measuring CSF secretion

CSF secretion was measured as described in Karimy et al. [41]. Animals were placed in a stereotaxic frame with ear bars. An incision was made along the midline of the scalp and a burr hole was made in the left parietal bone −0.8 mm posterior and 1.8 mm lateral to Bregma. A hollow screw was sealed into this position to guide the insertion of the lateral ventricle catheter. 

To expose the cisterna magna, the back of the neck was shaved, and a 1 cm incision made in the space between the occipital bone and the axis (C1 vertebrae). The suboccipital muscles were dissected from the occipital bones to expose the atlanto-occipital ligament. The neck was then rotated 90° on the ear bars so that its nose was pointing down. The ligament was resected to expose the underlying dura. The dura was then punctured (1 mm diameter) to access the cisterna magna. A polyethylene catheter (PE-20, Braintree Scientific, MA, USA) loaded with sterile, molecular grade mineral oil was inserted through the membrane and advanced 5 mm through the foramen of Magendie into the fourth ventricle. Mineral oil (100 μL) was infused into the fourth ventricle to occlude the aqueduct of Sylvius. The catheter was sealed into the cisterna magna using a sealant to prevent movement and unblocking of the aqueduct.

The left lateral ventricle was then cannulated using a 27 G neonatal lumbar needle (Becton, Dickinson and Company, NJ, USA) attached to silastic tubing (0.64 mm internal diameter; Axieo Specialties, Australia). The tubing began to fill with CSF upon cannulation and the distance (millimetres) travelled at 5 min intervals was recorded. We used the following equation to calculate the volume of CSF secreted:


$$\mathrm{volume}=\pi \;x\;{\mathrm{radius}}^{2}x\;\mathrm{distance}\;\left(\mathrm{mm}\right)$$

The rate of CSF secretion (μL/min) was calculated as the slope of the volume-time relationship.

### 2.5. Drug Administration

Animals received either 10 mg/kg of the TRPV4 antagonist RN1734 in dimethyl sulfoxide (DMSO; 100%) (*n* = 4), 10 mg/kg NKCC1 antagonist bumetanide in ethanol (100%; *n* = 3), or 1 mL/kg DMSO intraperitoneally (*n* = 3). After the three-hour testing period was complete, seven animals received 10 mg/kg acetazolamide in saline (0.9%) intravenously as a positive control for reduced CSF secretion.

### 2.6. Statistics

Statistical analyses were carried out using GraphPad Prism 7 (GraphPad Software, La Jolla, CA, USA). Comparisons between pre-drug and post-drug physiological variables were conducted for each treatment group using the paired Student’s *t*-test. Pre-drug, post-drug and post-acetazolamide CSF secretion were averaged across their relative recording periods to produce a single value for each period and a paired Student’s *t*-test was used to determine any significant differences. We used *p* ≤ 0.05 as a threshold for significance. Data are presented as mean ± standard deviation (SD).

## 3. Results

### 3.1. Exclusions

Six animals were excluded from the study due to absence of CSF flow from the cannulated ventricle into the ventricular catheter and tubing. One animal died prior to drug administration. All exclusions were prior to group assignment. Ten animals were included in the final study.

### 3.2. Physiological Parameters

Physiological parameters of each treatment group are presented in Table 1. There were no significant differences between any of the measured parameters at baseline and post-treatment administration in any of the treatment groups (Student’s *t*-test).

### 3.3. Acetazolamide and CSF Secretion

Acetazolamide reduced CSF secretion from 0.34 ± 0.08 µL/min at baseline to 0.17 ± 0.05 µL/min post-infusion when averaged across all groups, *n* = 7, t(6) = 4.29, *p* = 0.005.

### 3.4. NKCC1 Antagonism with Bumetanide

**Figure 2:**
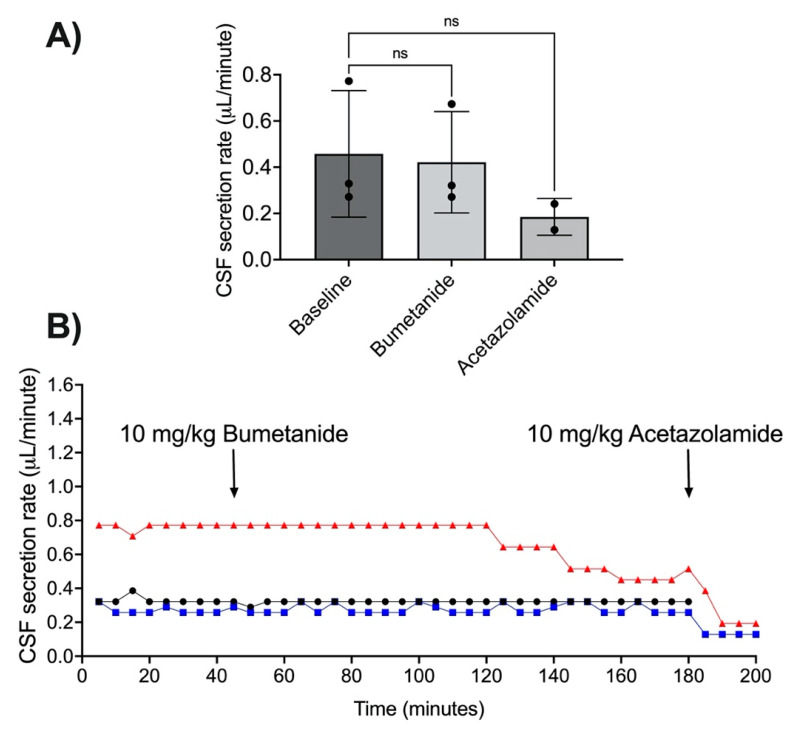
Effects of bumetanide on cerebrospinal fluid (CSF) secretion. (**A**) CSF secretion rate (mean ± SD; µL/min) for all animals at baseline, after administration of bumetanide, and after administration of acetazolamide. Points represent individual animals. (**B**) Individual CSF secretion rate plots over time in response to bumetanide and acetazolamide. Symbols and colours are distinct for each animal. ns = no significant difference, Student’s *t*-test compared with baseline.

Bumetanide did not change CSF secretion rate (*n* = 3, t(2) = 1.14, *p* = 0.37; Figure 2A,B). Average CSF secretion rate was 0.45 ± 0.27 µL/min at baseline. Average CSF secretion was 0.42 ± 0.22 µL/min after bumetanide administration. Two animals received acetazolamide after the recording period, this reduced CSF secretion to 0.19 ± 0.08 µL/min, a 40% reduction from baseline (*n* = 2, t(1) = 1.73, *p* = 0.33; Figure 2A,B). One animal did not receive acetazolamide as data were included from early in the study phase, prior to the introduction of the CSF secretion validation step.

### 3.5. TRPV4 Antagonism with RN1734

**Figure 3:**
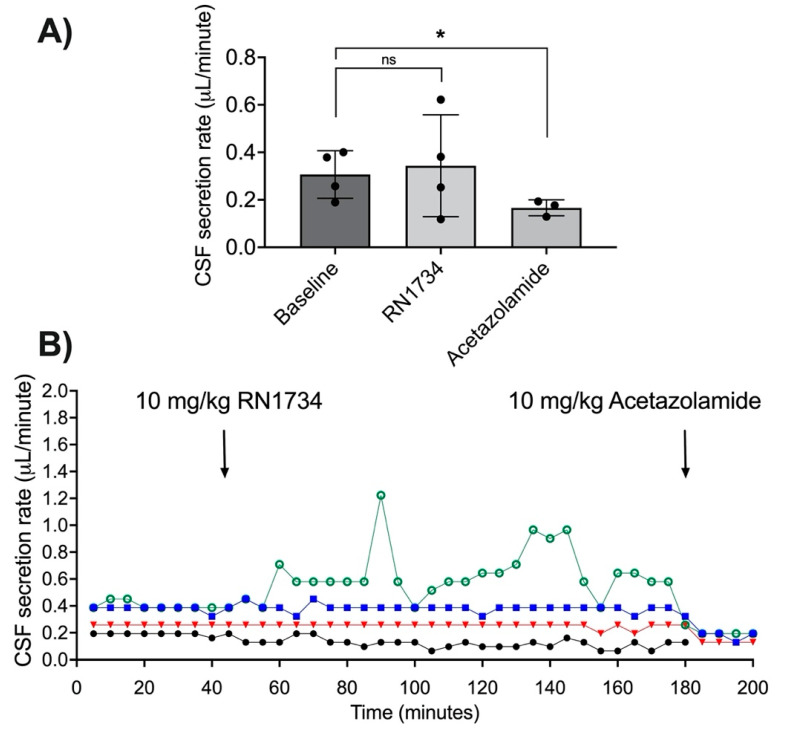
Effects of RN1734 on cerebrospinal fluid (CSF) secretion. (**A**) CSF secretion rate (mean ± SD; µL/min) for all animals at baseline, after administration of RN1734, and after administration of acetazolamide. Points represent individual animals. (**B**) Individual CSF secretion rate plots over time in response to RN1734 and acetazolamide. Symbols and colours are distinct for each animal. ns, not significantly different, * *p* ≤ 0.05, Student’s *t*-test compared with baseline.

The average CSF secretion rate did not change after RN1734 was administered (*n* = 4, t(3) = 0.58, *p* = 0.6, Figure 3A,B). The average baseline CSF secretion rate was 0.31 ± 0.1 µL/min. The average CSF secretion was 0.34 ± 0.21 µL/min after RN1734 administration. Individual responses to RN1734 were variable: CSF secretion did not change in two animals, reduced by 38% in one, and increased by 56% in another. Three animals received acetazolamide after the recording period, this reduced CSF secretion to 0.17 ± 0.03 µL/min, a 52% reduction from baseline (*n* = 3, t(2) = 7.07, *p* ≤ 0.02; Figure 3A,B). One animal did not receive acetazolamide as data were included from early in the study phase, prior to the introduction of the CSF secretion validation step.

### 3.6. DMSO as a Vehicle for RN1734 Administration

**Figure 4:**
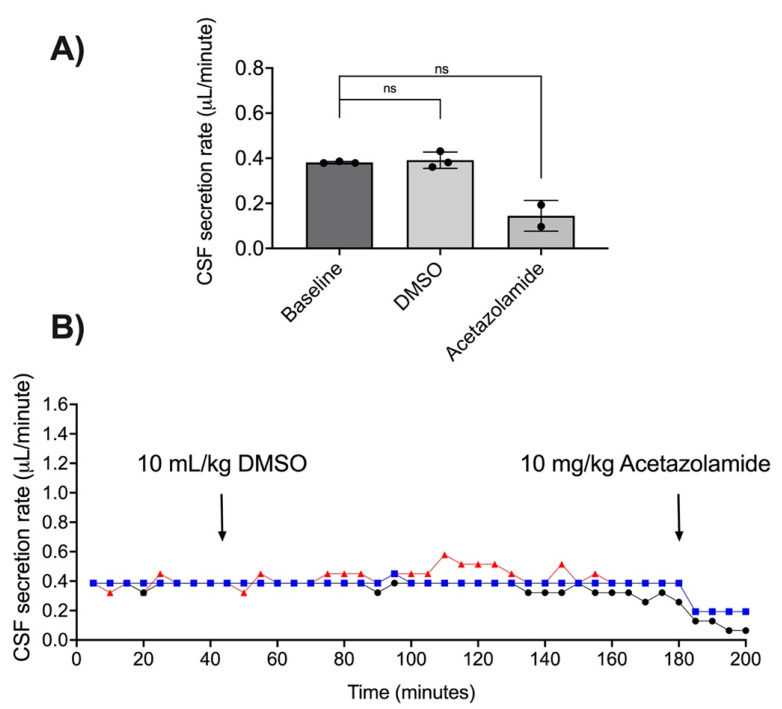
Effects of dimethyl sulfoxide (DMSO) on cerebrospinal fluid (CSF) secretion. (**A**) CSF secretion rate (mean ± SD; µL/min) for all animals at baseline, after administration of DMSO, and after administration of acetazolamide. Points represent individual animals. (**B**) Individual CSF secretion rate plots over time in response to DMSO and acetazolamide. Symbols and colours are distinct for each animal. ns = no significant difference, Student’s *t*-test compared with baseline.

DMSO did not alter the CSF secretion rate (*n* = 3, t(2) = 0.47, *p* = 0.69; Figure 4A,B). The average CSF secretion rate was 0.38 ± 0.01 µL/min at baseline. The average CSF secretion was 0.39 ± 0.04 µL/min after DMSO administration. Two animals received acetazolamide after the recording period, this reduced CSF secretion to 0.14 ± 0.07 µL/min, a 62% reduction from baseline (*n* = 2, t(1) = 5.32, *p* = 0.12; Figure 4A,B). One animal did not receive acetazolamide as data were included from early in the study phase, prior to the introduction of the CSF secretion validation step.

## 4. Discussion

In this study, we explored the potential of targeting NKCC1 and TRPV4 to control CSF secretion. Pharmacological therapies like these would be advantageous in neurological diseases where increased CSF secretion contributes to increased ICP. We used a direct approach for measuring CSF secretion and found that antagonism of NKCC1 and TRPV4 did not decrease CSF secretion rates in healthy rats naïve to neurological disease or injury. This suggests that these proteins are ineffective targets for controlling CSF secretion, at least in the absence of neuropathological mechanisms. 

Bumetanide and other loop diuretics acting on NKCC1, like furosemide, were previously used for the clinical management of elevated ICP [24,25]. However, they are not recommended in current clinical guidelines, which may be in part a reflection of the paucity of effective therapies and poor evidence-base. There is a shortage of recent original research to support the effects of bumetanide on CSF secretion, with few in vivo studies available from this century. Our study found that bumetanide administration had no effect on CSF secretion, acutely. One previous study reported a 49% decrease in CSF secretion after bumetanide administration to the lateral ventricles in mice [40]. Bumetanide inhibition of NKCC1 was also shown to decrease flow of CSF tracer in this study. Another older study reported a ~30% reduction in CSF secretion in dogs after bumetanide was added to the lateral ventricles [31]. However, CSF rates were variable between animals. These studies utilised tracer dilution techniques to determine CSF production, where tracer is infused into the lateral ventricles and collected at the cisterna magna. CSF secretion can be calculated by determining the tracer dilution in CSF. Tracer dilution techniques are an indirect measurement of CSF secretion and present several issues: (1) CSF flow is slow and occurs in a highly complex three-dimensional space, which probably never reaches a steady state, making this technique insufficient for studying the effects of pharmacological manipulation; (2) differences in CSF flow and tissue perfusion between animals are not considered, which limits the ability to determine the extent to which altered tracer dilution is explained by reduced CSF secretion, where reduced CSF flow in response to drug administration or respiratory changes may also impact tracer dilution; (3) CSF secretion was previously shown to decrease over time using this technique; (4) artefacts can be introduced to the data through minor variances in ICP or CSF outflow resistance [19,60,61]. We utilised systemic administration of bumetanide rather than intraventricular infusion to improve clinical relevance, Vogh et al. [19] previously found that systemic bumetanide injection did not alter CSF secretion in rabbits. Some have suggested that systemic injection of bumetanide may result in dissociation and poor absorption due to its low solubility in water. However, furosemide, an NKCC1 and carbonic anhydrase inhibitor, which also has a low water solubility, effectively reduces CSF secretion after systemic administration [19]. Further, bumetanide is detected in brain tissue after systemic administration of 10 mg/kg, the same dose used in this current study [58]. The same dose produces inhibition of NKCC1 in brain tissue and another study demonstrated that systemic bumetanide can reduce oedema following intracerebral haemorrhage after intraperitoneal injection [59,62]. Therefore, we would expect systemic bumetanide to reduce CSF secretion if this response was primarily regulated by the NKCC1 channel. In this study we used a direct method of measuring CSF secretion previously described by Karimy et al. [41], which allowed us to determine the effects of pharmacological agents in real-time. This method is not influenced by variances to CSF clearance and tissue perfusion and obtains relatively consistent CSF secretion values. Our findings provide further evidence that NKCC1 antagonism may be an ineffective target for managing CSF secretion. Further, the shortage of recent evidence in this area may reflect a publication bias in which negative results often go unreported. The conflicting findings of studies in this area highlights the need for increased investigation and reporting of results, regardless of outcome.

We hypothesised that TRPV4 antagonism would reduce CSF secretion, as previous studies of TRPV4 expression sites and ion transport activity have alluded to a regulatory role for the protein in CSF secretion [44,63]. Additionally, chronic TRPV4 antagonism is effective at reducing symptoms of hydrocephalus in rats, a disease characterised by the abnormal accumulation of CSF [55]. We report that acute antagonist administration did not decrease average CSF secretion rates. TRPV4 antagonism had a divergent effect in two rats: one rat had an increase (156% of baseline) and one rat had a decrease (62% of baseline) in CSF secretion after antagonist administration. However, we note that particularly for the animal with an increase in secretion, there were very large fluctuations in the secretion rate over the monitoring period. This is quite unusual for CSF secretion, which is generally quite consistent over time, even in the presence of significant systemic physiological perturbations [64]. A recent study found the presence of two additional glycosylated forms of TRPV4 in choroid plexus tissue, which have not yet been detected in other tissues expressing TRPV4 [55]. This suggests possible activation and inactivation of choroid plexus TRPV4, which could explain the variable responses found in our study. Additionally, chronic TRPV4 antagonism reduced cranial dimensions (as a measurement of CSF accumulation) in hydrocephalus rats but not in wild-type controls. The authors of the study conclude that activation of TRPV4, but not expression levels, may be altered in neurological diseases with increased CSF secretion, such as hydrocephalus [55]. These results suggest that different mechanisms of CSF regulation may exist under different conditions. We note that there are likely to be differences between chronic hydrocephalus and neurological diseases with acute ICP elevation. Our study was conducted in rats in the absence of neurological disease or injury; therefore, we cannot dismiss the possibility that TRPV4 antagonism has different effects on CSF secretion under pathological conditions. Chronic antagonism, rather than acute, may also present different observations. However, acute systemic administration of 5 mg/kg RN1734 effectively reduces expression of TRPV4 in the CNS [56]. Another study reported upregulation of NKCC1 in response to TRPV4 antagonism, which may present another source of variation [40]. Overall, our results indicate that TRPV4 is unlikely to be the sole regulator of CSF secretion under normal conditions.

Our direct approach for measuring CSF secretion was advantageous over tracer dilution techniques for measuring CSF secretion as values are less likely to be overestimated due to tissue uptake of tracer [41,65]. The technique we used involves blocking the cerebral aqueduct, which prevents normal CSF flow to the subarachnoid space. This allowed us to minimise any differences in CSF flow and clearance rates between animals. CSF secretion rates are likely to be underestimated as the average CSF secretion rate observed in our study was lower than previously described in rats using similar techniques (~0.37 μL/min vs. ~1 μL/min [41]). This was despite ensuring all animals used in the study were male Wistar rats over the age of 12 weeks, where adult CSF secretion rates are observed without the variation observed in younger rats [41]. Baseline CSF secretion rates were consistent in our study and were not age-dependent. Our method offers an in-animal comparison between baseline and drug administration which strengthens our study findings. The varied response to TRPV4 antagonism may have impacted our ability to detect average changes in CSF secretion rate, along with the low number of animals used for our study. We calculated that at least 13 animals per group would be required to detect a 30% change in CSF secretion (baseline 0.37 ± 0.15 μL/min baseline, 0.05 α-error probability, 80% power). We halted our study prior to obtaining these numbers due to the absence of a response relative to the consistent and robust effect seen with acetazolamide. 

## 5. Conclusions

We hypothesised that NKCC1 or TRPV4 antagonism would reduce CSF secretion rates in rats, as both proteins have been implicated in CSF secretion and have been targeted for mitigating ICP elevation and symptoms of increased cranial CSF volume. We conclude that acute antagonism of either NKCC1 or TRPV4 is ineffective at reducing CSF secretion in rats absent of neurological disease or injury. Further investigation of protein changes and antagonism could be explored in neurological disease where increased CSF secretion and ICP are observed before discounting the therapeutic potential of protein antagonism at these sites.

## Figures and Tables

**Table 1:** Physiological parameters before and after drug administration for each group. Comparisons between baseline and post-treatment were carried out using paired Student’s *t*-test.

	RN1734 (*n* = 3)		DMSO (*n* = 4)		Bumetanide (*n* = 3)	
	Baseline	Post-treatment	Baseline	Post-treatment	Baseline	Post-treatment
SpO_2_ (%)	97.5 ± 2.1	97.3 ± 1.5	97.7 ± 2.3	97.3 ± 2.08	98.0 ± 1.7	98.7 ± 0.6
Heart rate (BPM)	391 ± 54	403 ± 45	388 ± 13	391 ± 30	406 ± 30	411 ± 43
Mean arterial pressure (mmHg)	104.8 ± 3	102.5 ± 9.9	100 ± 11	100 ± 13.7	89.3 ± 7.2	84.0 ± 7
Respiratory rate (per minute)	66 ± 8	71 ± 4	69 ± 2	72 ± 4	70 ± 2	73 ± 2
paO_2_ (mmHg)	139 ± 47.6	136 ± 42.2	137 ± 30.9	158 ± 16.5	151 ± 49.3	238 ± 54.3
paCO_2_ (mmHg)	57.5 ± 4.9	57.7 ± 8.6	65.5 ± 6.6	62.9 ± 15.9	73.6 ± 22.6	64.4 ± 3.9
pH	7.31 ± 0.01	7.30 ± 0.03	7.23 ± 0.04	7.28 ± 0.09	7.21 ± 0.08	7.25 ± 0.02

## Data Availability

Data available upon request.
